# Detection of post-traumatic abdominal pseudoaneurysms by CEUS and CT: A prospective comparative global study (the PseAn study)—study protocol

**DOI:** 10.3389/fsurg.2023.1124087

**Published:** 2023-02-20

**Authors:** Francesco Virdis, Stefano Piero Bernardo Cioffi, Fikri Abu-Zidan, Elisa Reitano, Mauro Podda, Michele Altomare, Andrea Spota, Roberto Bini, Jayant Kumar, Osvaldo Chiara, Stefania Cimbanassi

**Affiliations:** ^1^ Trauma and Acute Care Surgery, ASST-GOM Niguarda, Milan, Italy; ^2^ The Research Office, College of Medicine and Health Sciences, United Arab Emirates University, Alain, United Arab Emirates; ^3^ Department of Translational Medicine, Division of General Surgery, Maggiore Della Carità Hospital, University of Eastern Piedmont, Novara, Italy; ^4^ Azienda Ospedaliero-Universitaria Cagliari, Cagliari, Italy; ^5^ University of Chicago Medical Center, Chicago, IL, United States

**Keywords:** trauma, ultrasoud diagnosis, follow up, abdominal trauma, contrast—enhanced ultrasonography

## Abstract

The success of non-operative management in trauma increased with the availability of new-generation CT scan machines, endoscopy, and angiography, becoming the standard of care in hemodynamically stable trauma patients with abdominal solid organ injuries, with a success rate of 78% to 98%. Post-traumatic pseudoaneurysms (PAs) can develop at any region of an injured artery and they may cause delayed bleeding in splenic or hepatic trauma, with an incidence in patients treated with NOM of 2%–27% and 1.2%–6.1% respectively. Diagnosis is made by angiography, contrast-enhanced computer tomography (CT), or Doppler Ultrasound (US) while the use of contrast-enhanced ultrasound (CEUS), has increased in recent years although few data are available about CEUS feasibility in the follow-up setting. The PseaAn study has been designed to assess the role of CEUS in the follow-up of abdominal trauma by defining its sensitivity, specificity and predictive values compared with abdominal CT scan. The PseAn study is a multi-centric international diagnostic cross-sectional study initiated by the Level I Trauma Center of the Niguarda Ca’ Granda Hospital in Milan, Italy. To study the role of CEUS in detecting post-traumatic splenic, hepatic, and renal PAs compared with the gold standard of CT with intravenous contrast at different follow-up time points, and whether it can replace CT scan in the follow-up of solid organ injuries, patients with OIS III and above will undergo a follow-up with both a CEUS and CT scan to detect post-traumatic parenchymal pseudoaneurysm within two to five days from injury. The use of CEUS in the follow-up of abdominal trauma follow-up (particularly blunt trauma) has increased, to minimise the use of ionizing radiation and contrast media and encouraging results have been published during the last decade showing that CEUS is an accurate technique for evaluating traumatic lesions of solid abdominal organs. Conclusions We think that CEUS, which is underused worldwide, is a useful and safe tool that may replace CT scan in follow-up with the major advantage of reduced radiation. Our current study may give stronger evidence to support this view.

## Introduction

Nonoperative management (NOM) is the standard of care in hemodynamically stable trauma patients with abdominal solid organ injuries, with a success rate of 78% to 98% (1–4).

Trans-catheter angioembolization (AE) has reduced the failure of NOM even in patients with high-grade injuries. NOM is less likely to fail in liver injuries than in splenic or kidney injuries (5–7).

The success of NOM increased with the availability of new-generation CT scan machines, endoscopy, and angiography. This reduced surgery-related morbidity and increased spleen, kidney, and liver salvage rates (4).

Delayed bleeding during NOM, which occurs in up to 25% of splenic, 4% of hepatic, and 9% of renal injuries, is a life-threatening complication that needs urgent intervention (3, 8, 9).

Pseudoaneurysms (PAs) can develop at any region of an injured artery but commonly occur in the hepatic or splenic artery branches. Penetrating mechanism is more often associated with renal pseudo aneurysm compared with blunt trauma (10, 11).

Post-traumatic PAs may cause delayed bleeding in splenic or hepatic trauma, with an incidence in patients treated with NOM of 2%–27% and 1.2%–6.1% respectively (1, 12–14).

Diagnosis is made by angiography, contrast-enhanced computer tomography (CT), or Doppler Ultrasound (US) (15).

The use of the contrast ultrasound technique, known as contrast-enhanced ultrasound (CEUS), has increased in recent years. To date, the main CEUS method used in clinical settings is untargeted microbubbles, injected intravenously into the systemic circulation in a small bolus. While the microbubbles will endure in the systemic circulation, ultrasound waves are directed at the area of interest. A software operating at low mechanical index analyzes the resonance signals from the contrast agents which allows for performing all the vascular phases in real-time (16–18).

CEUS use was first described in 2008 in pediatric blunt abdominal trauma (19) and used in 2013 as a screening tool to identify traumatic PAs in adult patients, with a 75% sensitivity and 100% specificity, compared with CT scan in 63 adults (20).

It was useful in mild isolated abdominal trauma which can assess the grade of solid organ injuries similar to CT (21). Few data about CEUS feasibility in the follow-up setting are available. Manetta et al. highlighted the role of CEUS in the follow-up of patients with low-grade hepatic or splenic injuries and other authors found it superior to unenhanced CT in evaluating abdominal pathologies including renal impairment (22–25).

Moreover, few authors described CEUS to be as accurate as CT scan in the identification of the damage and the healing process, which has less radiation in young patients and pregnant females (22).

The PseaAn study will assess the role of CEUS in the follow-up of abdominal trauma by defining its sensitivity, specificity and predictive values compared with abdominal CT scan.

## Aim

We aim to study the role of CEUS in detecting post-traumatic splenic, hepatic, and renal PAs compared with the gold standard of CT with intravenous contrast at different follow-up time points, and whether it can replace CT scan in the follow-up of solid organ injuries.

## Methods and design

Patients with OIS III and above will undergo a follow-up with both a CEUS and CT scan to detect post-traumatic parenchymal pseudoaneurysm within two to five days from injury. Follow-up over five days post-admission will be included if they were performed within the same hospitalization. Institutions enrolled are those which use CEUS within their clinical practice keeping up with the observational nature of the study. Furthermore, the study will prospectively define the incidence and outcome of traumatic parenchymal PAs in blunt and penetrating splenic, hepatic, and renal trauma as detected by CT scan. This will help us define the indications for a systematic follow-up as well as the criteria for intervention in post-traumatic PAs.

The PseAn study is a multi-centric international diagnostic cross-sectional study initiated by the Level I Trauma Center of the Niguarda Ca’ Granda Hospital in Milan, Italy. Institutions are recruited internationally. The Steering Committee is composed of trauma experts including delegates from the World Society of Emergency Surgery (WSES) which endorsed the study.

To avoid bias, two different radiologists will independently report the two procedures, each unaware of the other's outcome. Furthermore, radiologists selected in each Institution to be part of the study should be competent experts in the study area, defined as Consultant Radiologist level with good confidence in performing the procedures independently.

This is important to minimise the operator learning curve's effect (Trauma CT and CEUS).

## Inclusion and exclusion criteria

Consecutive adult patients (18 years old and above) with proven medium/severe (grade III and above) blunt or penetrating splenic and/or liver and/or renal trauma as shown by a CT scan and classified according to the American Injury Scale American Association for Surgery (AAST) Organ Injury Scale (OIS) will be included (26).

Patients who underwent surgery without NOM and patients who refuse to participate will be not considered for this study.

## Study periods

Provided the ethical approval, the promotion of the Psean Study and recruitment of Institutions will start from the 1st of October 2022 until the completion of data collection, according to the calculated sample size. The online database will be available for entering the data from 01/10/2022 to 31/10/2023. Data verification and analysis will follow.

## Data collection

National committees will lead the study in participating countries, with overall coordination provided by the study steering committee. The PseAn study involves clinical centres from different countries worldwide. In each centre, the coordinator will collect epidemiological, clinical, and surgical data on a case report form (CRF) that will be completed through a questionnaire by accessing a protected database.

The protocol for data collection is shown in Supplementary Appendix S1. The link for accessing the completion of the CRF will be sent by email, to the Main Lead of each participating centre. The PseaAn Steering Committee developed the database using web-based and remote discussions, after identifying the key components and topics to be included. Online questions and response items in the Google Forms database are available by accessing the link https://lnkd.in/d6U7wW33.

Data will be collected contemporaneously on a dedicated server that allows collaborators to enter and store data in a secure system. Data will be anonymous and no patient unique identifiers (name, date of birth, address, telephone number, etc.) will be collected.

## Statistical analysis

### Sample size

Because of the lack of literature where the two diagnostic methods are compared providing an interquartile range, a sample size for an unknown population has been chosen. The sample size for unknown populations has been derived according to the formula *N* = Z2 × p (1-p)/e2, setting standard deviation (SD) at 50%, confidence interval at 95%, and Z-score at 1.65. The minimum sample size was 385 patients with a sampling error of ±5%.

### Statistical comparisons

Cronbach alpha which measures internal consistency will be used to compare the radiologists' reports. To reduce the impact of confounding factors, the propensity score will be calculated to estimate relevant clinical effects adjusted for given confounders like age, sex, and mechanism of injury. Continuous data will be compared using Student *t*-test and Mann Whitney according to the distribution of data. As appropriate, the chi-squared test or Fisher's exact test will be used for categorical data analysis. A value of *P* < 0.05 will be considered statistically significant. The sensitivity, specificity, positive, and negative predictive values and Likelihood ratio of the CEUS will be calculated using CT scan as the gold standard.

## Ethical considerations

The research protocol and performance of the study will adhere to the standards outlined in the Declaration of Helsinki and Good Epidemiological Practices. Centres will be responsible for Ethics Committee approval depending on their local policy. Informed consent will be signed by the participants or their caregivers to collect their anonymous data for this study. The consent form will include all information and technical details of contrast CT and CEUS and possible related side effects.

## Publication policy

The Main Lead and two Collaborators from each centre will appear as Co-authors in the final paper.

Data collected from the PseudoStudy study will be published irrespective of the findings and the results will be published on ClinicalTrials.Gov (ID: NTC05627908).

## Discussion

To date, there are no agreed standardized guidelines on follow-up of splenic, hepatic, and renal trauma, nor clear indications for angioembolization in post-traumatic pseudoaneurysm.

A panel of experts, in collaboration with the World Society of Emergency Surgery, recently published a consensus on follow-up strategies for patients with splenic trauma managed non-operatively (27).

Methods and timing of follow-up and treatment of post-traumatic PAs are variable. Some authors do not perform imaging follow-up at 48 to 72 h after admission in patients with low-grade injuries (28), while others perform routine follow-up CT for detecting delayed PAs and treat them with early embolization before rupture (4, 29).

Sabe et al. suggested a protocol, which incorporated the initial use of selective AE for patients having a high risk for NOM failure and developing Pas, while others performed AE for PAs of solid organs of 15 mm diameter or more (6, 29).

Different authors suggested that small splenic PAs do not need intervention because they can spontaneously occlude in 2 to 10 days after detection, especially when they are less than 10 mm (29–31).

The current literature is not conclusive regarding the use of AE for single vascular abnormality including contrast blush, pseudo-aneurysms, and arterio-venous fistula in minor and moderate injuries (32).

The decision and timing of follow-up after admission is often based on the CT findings on admission; low-grade injuries (Organ Injury Scale I and II) are usually not followed-up. Recent guidelines have advised considering repeating the CT scan during admission in patients with moderate and severe lesions (OIS III and above), decreasing hematocrit, the presence of vascular anomaly, underlying pathology, coagulopathy, or neurologically impairment (32).

The timing and type of imaging follow-up [CT or Ultrasound (US)] have not been agreed on and it is usually based on clinical judgment (32).

The use of CEUS in the follow-up of abdominal trauma follow-up (particularly blunt trauma) has increased, to minimise the use of ionizing radiation and contrast media (21).

Encouraging results have been published during the last decade showing that CEUS is an accurate technique for evaluating traumatic lesions of solid abdominal organs that can identify active bleeding and vascular lesions and monitor patients who undergo NOM (33).

CEUS significantly improves radiological evaluation of hepatic, splenic and renal injuries which correlates well with contrast CT scan, compared with traditional ultrasound. It can also identify minor blood flow and delineate vascular structures in detail (34), and it seems to be a valid adjunct or alternative imaging modality to contrast-enhanced CT in pediatric blunt abdominal trauma because of the reduction of exposure to ionizing radiation (35).

CEUS represents a valid alternative to contrast CT scan, particularly in patients with contraindications to CT contrast agents, in children and pregnant women.

CEUS is an effective, quick, and cost-effective imaging tool without ionizing radiation.

We think that CEUS, which is underused worldwide, is a useful and safe tool that may replace CT scan in follow-up with the major advantage of reduced radiation and costs. Our current study may give stronger evidence to support this view.
