# Supplementary material for: Detection of post-traumatic abdominal pseudoaneurysms by CEUS and CT: A prospective comparative global study (the PseAn study)—study protocol
Source: Front Surg. 2023 Feb 20;10:1124087. doi: 10.3389/fsurg.2023.1124087 (PMC9986536; doi:10.3389/fsurg.2023.1124087)
Supplement: Supplementary file 1 [file Datasheet1.pdf]

Follow-up of pseudoaneurysms after abdominal trauma: comparison between CEUS and CT scan in the evaluation of hepatic, splenic and renal post-traumatic pseudoaneurysms.

The PseAn Study

*Required

1. Name Local Lead *

____

2. Institution *

____

3. Level Trauma Centre *

Mark only one oval.

- ( ) I
- ( ) II
- ( ) III
- ( ) Other

Patient Details

4. Sex *

Mark only one oval.

- ( ) M
- ( ) F

5. Age *

Mark only one oval.

- ( ) 0-17
- ( ) 18-39
- ( ) 40-64
- ( ) 65-74
- ( ) >75

6. Comorbidities

Tick all that apply.

- [ ] COPD
- [ ] Diabetes
- [ ] Hypertension
- [ ] Cardiovascular Disease

7. Anticoagulation *

Mark only one oval.

- ( ) Yes
- ( ) No

Trauma

8. Mechanism of injury *

Mark only one oval.

- ( ) Blunt
- ( ) Penetrating

9. Spleen injury OIS

Mark only one oval.

- I
- II
- III
- IV
- V

10. Kidney injury OIS

Mark only one oval.

- I
- II
- III
- IV
- V

11. Liver injury OIS

Mark only one oval.

- I
- II
- III
- IV
- V
- VI

Follow-up

CEUS = contrast-enhanced ultrasound
PAs = pseudoaneurism
AE = angioembolization

12. Timing CT scan follow up *

Mark only one oval.

- 24h
- 48-72h
- >72h

13. Timing CEUS scan follow up *

Mark only one oval.

- 24h
- 48-72h
- >72h

14. PAs at CT follow-up *

Mark only one oval.

- Yes
- No

15. PAs at CEUS follow-up *

Mark only one oval.

- Yes
- No

16. Site of PAs

Mark only one oval.

- Liver
- Spleen
- Kidney

17. Size of PAs (CT/CEUS)

Mark only one oval.

- 1-5 mm
- 6-10 mm
- > 10 mm

18. Angioembolization of PAs

Mark only one oval.

- Yes
- No

19. Failure of AE

Mark only one oval.

- Yes
- No

20. Length of hospital stay (days) *

Mark only one oval.

- up to 7
- 8-15
- >15

21. Post Traumatic Morbidities

____

22. Post Traumatic Mortality *

Mark only one oval.

- Yes
- No

This content is neither created nor endorsed by Google.

Google Forms
